# Characterization of diseased primary human hepatocytes in an all-human cell-based triculture system

**DOI:** 10.1038/s41598-024-57463-7

**Published:** 2024-03-21

**Authors:** Justin J. Odanga, Sharon M. Anderson, Erick K. Breathwaite, Sharon C. Presnell, Edward L. LeCluyse, Jingsong Chen, Jessica R. Weaver

**Affiliations:** 1 Institute of Regenerative Med., LifeNet Health, 1864 Concert Dr., Virginia Beach, VA USA; 2 Research and Development, LifeNet Health LifeSciences, 6 Davis Dr., Research Triangle Park, NC USA; 3 LifeSciences Product Development, LifeNet Health, 1864 Concert Drive, Virginia Beach, VA 23453 USA

**Keywords:** Liver, Hepatocytes, Human, Disease, NASH, Cell biology, Diseases

## Abstract

Liver diseases, including NAFLD, are a growing worldwide health concern. Currently, there is a lack of suitable in vitro models that sustain basic primary human hepatocyte (PHH) morphology and functionality while supporting presentation of disease-associated phenotypic characteristics such as lipid accumulation and inflammasome activation. In TruVivo, an all-human triculture system (hTCS), basic metabolic functions were characterized in PHHs isolated from normal or diseased livers during two-weeks of culture. Decreases in albumin and urea levels and CYP3A4 activity were seen in diseased-origin PHHs compared to normal PHHs along with higher CYP2E1 expression. Positive expression of the macrophage markers CD68 and CD163 were seen in the diseased PHH preparations. Elevated levels of the pro-inflammatory cytokines IL-6 and MCP-1 and the fibrotic markers CK-18 and TGF-β were also measured. Gene expression of *FASN, PCK1,* and *G6PC* in the diseased PHHs was decreased compared to the normal PHHs. Further characterization revealed differences in lipogenesis and accumulation of intracellular lipids in normal and diseased PHHs when cultured with oleic acid and high glucose. TruVivo represents a promising new platform to study lipogenic mechanisms in normal and diseased populations due to the preservation of phenotypic differences over a prolonged culture period.

## Introduction

Obesity and chronic liver injury are major global health concerns with increasing prevalence. Non-alcoholic fatty liver disease (NAFLD) represents a substantial portion of the healthcare burden in Western countries afflicting 20 to 30% of the general population^1,2^. The spectrum of NAFLD is wide ranging and includes benign simple steatosis to nonalcoholic steatohepatitis (NASH), fibrosis, cirrhosis, and hepatocellular carcinoma (HCC)^3^. Causes of drug-and chemical-induced steatohepatitis and metabolic syndrome have been difficult to study due to the lack of suitable in vitro human cell-based models that maintain hepatocytes from diseased donor tissues^4,5^. Some 3D model systems, including spheroids and liver-on-a-chip, are highly complex, difficult to reproduce, and costly^4,5^. Animal disease models have been used to fill this gap; however, the interspecies differences do not accurately model human disease due to the use of artificial induction regimes and/or the lack of consistent human-relevant pathogenesis^4,6^. With the limitations of existing animal models, there is a need for improving human liver model systems that sustain hepatic cells from diseased tissues and better retain key elements of human NAFLD.

One complication of in vitro models is the lack of an accurate and consistent diseased phenotype. A model system is needed that maintains diseased characteristics including lipid accumulation and inflammation for extended culture periods. In the early stages of NAFLD, accumulation of lipids in hepatocytes by excess dietary fats, absorption of free fatty acids (FFAs), lipogenesis, or insufficient fatty acid oxidation can result in steatosis and insulin resistance^7^. Once steatosis begins, reactive oxygen species (ROS) are generated, leading to activation of immune cells resulting in inflammation and liver injury, the second phase of NAFLD^8,9^. These immune cells, including macrophages, secrete pro-inflammatory cytokines leading to increased levels of collagen I, α-smooth muscle actin and transforming growth factor-β (TGF-β). The resulting deposition of extracellular matrix and fibrosis is the third stage of NAFLD. Ideally, a diseased model system should progress to display features from all 3 stages of NAFLD.

Recently, TruVivo, an all-human cell-based triculture system (hTCS), was developed to represent a normal liver system^10^. The basic platform consists of 3 different cell types including cryopreserved primary human feeder cells (FCs), a mix of stromal cells and endothelial cells, and cryopreserved primary human hepatocytes (PHHs). In this hTCS, PHHs from healthy donor tissues maintained histotypic morphology and formed anastomosing networks of bile canaliculi with tight and gap junctions for greater than 42 days with no ECM overlay. Moreover, the cultures showed stable basic hepatocyte functions, including albumin and urea production and cytochrome P450 (CYP) 3A4 activity. In this study, disease-origin PHHs were applied to the hTCS (DhTCS) to explore the feasibility of retaining relevant disease features in the model. For these studies, PHHs were selected from tissue donors with known medical history of NAFLD and histopathologic confirmation of disease.

Furthermore, a prototypical Farnesoid X Receptor (FXR) agonist Obeticholic Acid (OCA) was applied in the hTCS cultured in a high FFA/high glucose medium, a type of lipogenic (lipo) medium shown to induce lipogenesis while not being toxic to cells, to determine the capacity of the hTCS to detect perturbation and/or modulation of key NAFLD-associated biomarkers^11,12^. In vivo murine models have shown reduced lipogenesis, inflammation, and fibrosis with OCA treatment. Likewise, cultures of human hepatocytes, hepatic stellate cells, and/or macrophages in medium containing FFAs, including oleic acid, exhibited less lipid accumulation, decreased pro-inflammatory marker secretion, and decreased collagen biosynthesis with OCA treatment^13,14^.

In general, PHHs from diseased liver tissues in the DhTCS retained a stable phenotype and function for extended culture periods resulting in reduced hepatic functions of albumin synthesis, urea production, and CYP3A4 activity when compared to healthy-origin PHHs. Disease-relevant features included lipid accumulation, CYP2E1 upregulation, and presence of Cytokeratin 18 (CK-18) and TGF-β in the DhTCS. Induction of steatotic change and decreased hepatic functionality occurred upon exposure to lipogenic medium and was modulated by OCA. The presence and maintenance of these diseased characteristics suggests the potential of the DhTCS to be a suitable platform for development of liver disease models.

## Materials and methods

### All-human triculture system

The hTCS was set up as previously described, including the medias, cell types, and expected functional performance^15^, also described in US 11,535,827. 300,000 primary human hepatocytes and 50,000 primary human FCs were seeded per well. FC only wells were seeded using 50,000 cells per well. The FC only wells were cultured under the same experimental conditions as the hTCS and DhTCS. All methods were performed in accordance with the guidelines and regulations of LifeNet Health’s ethics committee. These methods were approved by LifeNet Health’s ethics committee. Informed consent was obtained for all donor tissue for research purposes by LifeNet Health. PHHs have been designated as normal or diseased based on a histopathologic assessment of the tissue of origin by a board-certified pathologist, using the standard NASH CRN Scoring System^16^. Formalin-fixed paraffin-embedded tissue sections were stained with H&E (Supplementary Fig. S1) and Masson’s Trichrome (Supplementary Fig. S2) and assessed for steatosis, lobular inflammation, and hepatocyte ballooning to form the NAFLD Activity Score (NAS). Fibrosis was scored separately on a 4-point scale according to the classification as described by Kleiner et al.^16^. Tissues with a NAS of ≥ 4 were designated “Diseased” (n = 4) while those with NAS of ≤ 3 were categorized as “Normal” (n = 7) (Supplementary Table S1).

### Morphological assessment, PHH attachment, and percent confluency

To morphologically assess the cells, they were imaged on the designated days using a BX41 microscope (Olympus, Tokyo, Japan) or Zeiss Observer.Z1 fluorescent microscope (Zeiss, Dublin, CA). The previously described method was used to determine PHH attachment and percent confluency^15^.

### Albumin and urea assays

Supernatant was collected on the indicated days for measurement of albumin and urea. Samples were run in duplicate. The concentration of albumin was determined using an ELISA assay (Abcam, Cambridge, MA) and performed according to the manufacturer’s instructions. Urea was measured by a colorimetric kit (Stanbio, Boerne, TX) and performed according to the manufacturer’s instructions.

### Bicinchoninic acid assay (BCA)

The BCA assay was performed with supernatant collected on the designated day. Samples were run in duplicate. Total protein concentration was determined using a kit (Thermo Fisher, Waltham, MA) and performed according to the manufacturer’s instructions.

### CYP3A4 assay

To induce CYP3A4 enzyme, cells were treated with 25 µM Rifampicin (Sigma, St. Louis, MO) in supplemented culture medium at 37 °C for 48 h prior to measuring CYP3A4 enzyme activity on the specified day. Samples were run in duplicate. The P450-Glo Assay kit was used to detect CYP3A4 enzyme activity (Promega, Madison, WI). After 48 h, the medium was removed, and the cells were washed with DMEM (no phenol red) (Thermo Fisher). Cyp-Luciferin-IPA stock was then added and incubated at 37 °C for 30 min. The supernatant was then collected, and the assay was performed according to the manufacturer’s instructions.

### Gene expression

Cells were lysed using RLT buffer (Qiagen, Germantown, MD). RNA was then isolated using the RNeasy kit (Qiagen) as per the manufacturer’s instructions. cDNA was prepared using the PrimeScript RT reagent kit (Takara Bio, Shiga, Japan) in a 30 µL volume reaction containing 6 µL 5X PrimeScript RT Master Mix, 20 µL RNA, and 4 µL ddH_2_O. qRT-PCR reactions contained 10 µL QuantiNova 2 × SYBR Green Master Mix (Qiagen), 2 µL ROX reference dye (1:10 dilution; Qiagen), 2 µL designated primer set (10 pM), 1 µL cDNA, and 5 µL ddH_2_O for a final volume of 20 µL. Primers used for *Glyceraldehyde 3-Phosphate Dehydrogenase* (*GAPDH)*, *Fatty Acid Synthase* (*FASN*), *Phosphoenolpyruvate Carboxykinase 1* (*PCK1*), *Glucose-6-Phosphatase Catalytic Subunit* (*G6PC), Interleukin-6 (IL-6), Monocyte Chemoattractant Protein-1 (MCP-1), Cytokeratin-18 (CK-18),* and *Transforming Growth Factor-β (TGF-β)* are listed in Supplementary Table S2 (Thermo Fisher). Taqman primers were used for *CD68* and *CD163* (Thermo Fisher). PCR amplification was done on a StepOne Plus Real Time PCR System (Applied Biosystems) using the following program: Step (1): 02:00 min at 95 °C; Step (2) 00:05 s at 95 °C; Step (3) 00:10 s at 60 °C. Repeat steps 2 and 3 for 40 cycles. Data was analyzed with StepOne software (version 2.3) (Applied Biosystems) and Microsoft Excel. Gene expression was normalized to the housekeeping gene *GAPDH* and analyzed using the 2^-ΔΔC^_T_ method^17–20^. It is acknowledged that the use of *GAPDH* as the housekeeping gene could lead to the exclusion of certain data sets. Data is presented as fold change relative to Feeder Cell (FC) expression or PHHs cultured in standard medium.

### Immunofluorescence and quantitation

Cells were fixed with a fixation solution (eBioscience, San Diego, CA) for 30 min at room temperature. They were then washed two times with 1X Permeabilization (eBioscience), and primary antibody was added at 4 °C overnight. The following antibodies were used: (1) CYP2E1 at 1:100 (Abcam, ab28146); (2) CD68 at 1:100 (Abcam, ab955); (3) CD163 at 1:100 (Abcam, ab87099); (4) anti-Monocyte Chemoattractant Protein-1 (MCP-1) at 1:100 (Abcam, ab9669); (5) anti-Interleukin-6 (IL-6) at 1:100 (Abcam, ab233706); (6) anti-CK-18 at 1:100 (Abcam, ab24561); and (7) anti-TGF-β at 1:100 (Abcam, ab92486). Cells were washed twice, and secondary goat anti-mouse IgG Alexa Fluor 488 conjugated antibody (Thermo Fisher) or secondary goat anti-rabbit IgG Alexa Fluor 555 conjugated antibody (Thermo Fisher) were added at a 1:500 dilution for 30 min (mins) at 4 °C. Cells were then washed twice, and Fluoromount-G mounting medium with DAPI (Invitrogen, Waltham, MA) was added for 20 min at room temperature. Images were taken using a Zeiss Observer.Z1 fluorescent microscope. Images were acquired with Z-stack using a Zeiss ApoTome.2 on the 10X objective lens.

The following method was used to quantitate fluorescent signal. Five images were taken from a pre-determined location for each sample well. Each well was focused in the 5X objective, followed by capturing images at the 10X objective. Exposure for DAPI, dsRed, and GFP was set at 300 ms, 1000 ms, and 2000 ms, respectively. Images were processed in ZEN software to remove fluorescent background by adjusting image grey values. All fluorescent channels were set using the same grey values determined for their specific filter. Individual and merged images were exported and opened in Image J. Individual channels were measured for fluorescent intensity using the “measure” function in ImageJ set to record integrated density.

### Luminex

Supernatant was collected on the indicated days for measurement of IL-6, MCP-1, CK-18, and TGF-β. Samples were run in duplicate. The concentration of each protein was determined using a Luminex assay (R&D Systems, LXSAHM-06, Minneapolis, MN) and performed according to the manufacturer’s instructions. The plate was read on Luminex XMAP Magpix. Luminex Xponent software version 4.3 was used to analyze the samples.

### Glucose, oleic acid, and OCA treatment

In the indicated experiments, cells were treated daily with lipo medium which included 25 mM glucose (Sigma) and 320 µM Oleic acid (Sigma, O3008) beginning on day 3 of the culture period and ending on day 17. “Day 1” is defined as 24 h after culture in the lipo medium. “Day 14” is the last day of culture in either standard medium or lipo medium or day 17 of the overall culture period. When indicated, 6α-ethyl-chenodeoxycholic acid (OCA) (AdipoGen Life Sciences, San Diego, CA) was added daily to the cells at a final concentration of 0.5 µM throughout the lipo medium culture period^14^.

### Nile red staining

Cells were washed with 1X DPBS (–Ca^++^/–Mg^++^) (Thermo Fisher) three times, and then Nile Red was added at a 1:500 dilution (Abcam). After fifteen mins at 37 °C, cells were washed twice with 1X DPBS (–Ca^++^/-Mg^++^) and then imaged on an EVOS FL cell imaging system (Thermo Fisher) using a 10X or 20X objective. Nile Red staining was quantified by determining the densitometric fluorescence value (red channel) using Image J.

### 5-(and-6)-carboxy-2’, 7’-dichlorofluorescein diacetate staining

To visualize bile canaliculi formation, cells were rinsed twice with 1X DPBS (–Ca^++^/–Mg^++^) (Thermo Fisher). Fresh medium plus 5 µM 5- (and-6)-Carboxy-2’, 7’-Dichlorofluorescein Diacetate (CDFDA) (Thermo Fisher) were added to the cells and incubated for 20 min at 37 °C. Cells were then washed twice with 1X DPBS (–Ca^++^/–Mg^++^), and DMEM (no phenol red) (Gibco) was added to the cells. Images were taken with an EVOS FL cell imaging system (Thermo Fisher) using a 10X or 20X objective.

### Calculating PHH attachment and statistical analysis

Images shown are from representative donor lots. For albumin, urea, and CYP3A4 measurements, values were normalized to the determined number of attached PHHs as described above except when noted otherwise^15^. Significance was calculated in MiniTab (State College, PA) using either Student’s *t* test or one-way ANOVA with Tukey post hoc testing to determine statistical significance with 95% confidence and *p < 0.05 for statistical significance. The Student’s t-test was used to calculate significance when comparing two groups, while the ANOVA with Tukey post hoc testing was used when comparing 3 groups or more.

## Results

### Morphology and functionality of normal versus diseased PHHs

**Figure 1:**
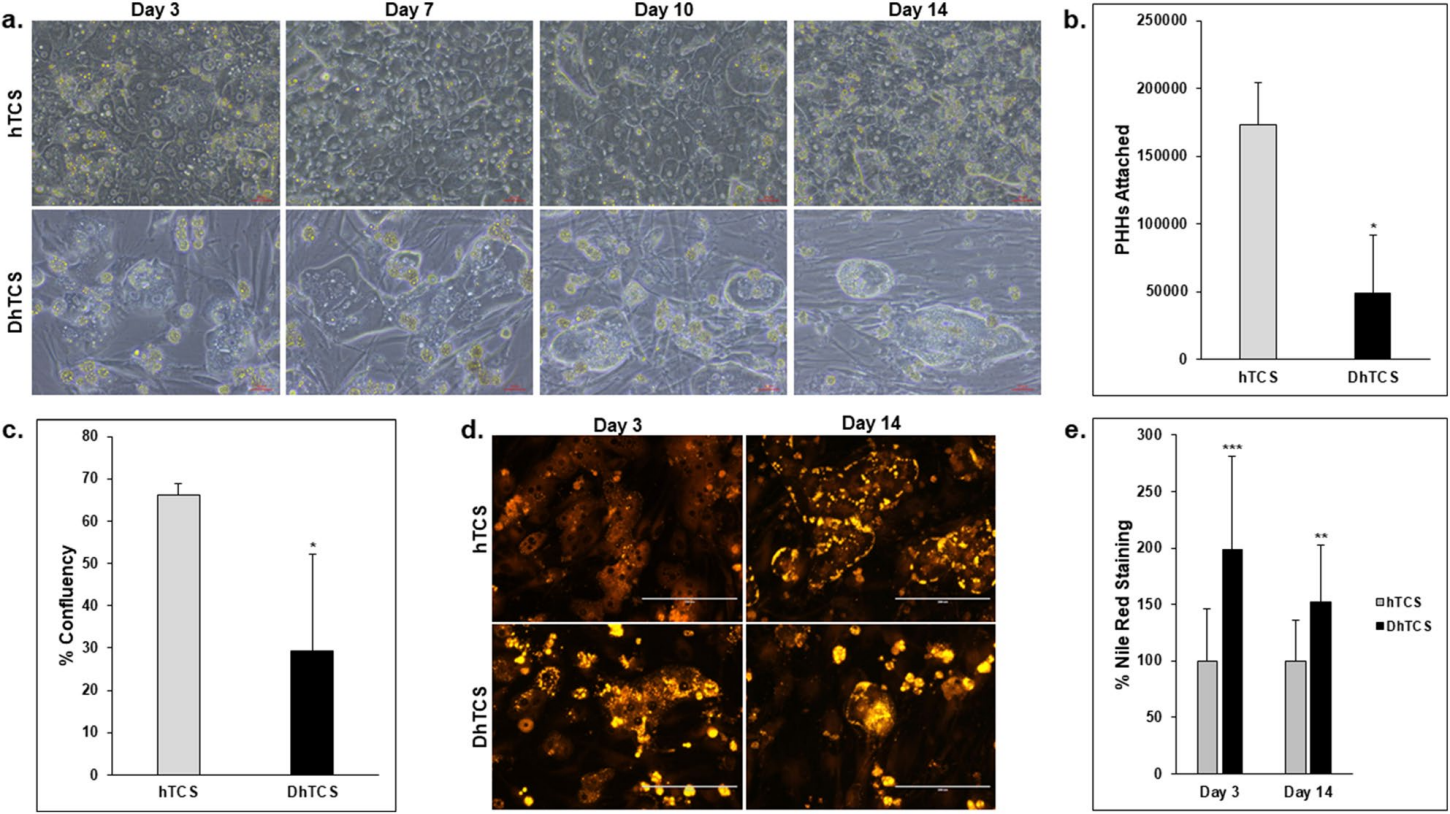
Differences in attachment and lipogenesis between normal and diseased PHHs in the hTCS and DhTCS. (**a**) Representative images of normal and diseased PHHs on days 3, 7, 10, and 14 in the hTCS and DhTCS. Magnification 20X. Scale bar = 50 µm. (**b**) The number of PHHs attached (n = 5 images per donor, n ≥ 3 donors per condition) and (**c**) percent confluency were determined in the hTCS and DhTCS on day 14 (n = 5 images per donor, n ≥ 3 donors per condition) (Student’s t-test). (**d**) Nile Red staining and (**e**) quantification in the hTCS (gray bars) and DhTCS (black bars) PHHs on days 3 and 14 (n ≥ 2 images per donor, n ≥ 2 donors per condition) (One-way ANOVA plus Tukey). Magnification 20X. Scale bar = 200 µm. *p ≤ 0.05, **p ≤ 0.01, ***p ≤ 0.001 to hTCS PHHs. Error bars represent standard deviation.

Based on the histopathology of the liver, donors were designated as normal (NAS score ≤ 3) versus diseased (NAS score of ≥ 4). Morphological differences were observed between the normal and diseased lots when cultured in the hTCS or DhTCS on days 3, 7, 10, and 14 (Fig. 1). Normal and mild to moderately diseased PHHs had a cuboidal shape and formed hepatocyte colonies. Visual differences in hepatocyte morphology were observed in the severely diseased PHHs (Fig. 1a). There were significant differences in PHH attachment (Fig. 1b) and percent confluency (Fig. 1c) between these two groups. Diseased PHHs attached with significantly less efficiency compared to normal PHHs (48,779 ± 42,703 vs 173,614 ± 31,080 PHHs). The confluency was significantly different between normal and diseased PHHs (66.1% ± 2.7 vs 29.2% ± 22.9). Diseased PHHs appeared to be more spread out and less compact when compared to normal PHHs and to have greater lipid accumulation compared to normal PHHs (Fig. 1d). Intracellular lipid content was doubled in diseased PHHs on day 3 (198.8% ± 82.8) compared to normal PHHs (100% ± 46.4) (Fig. 1e). A decrease in staining was measured from day 3 to 14 (151.9% ± 50.6) in diseased PHHs; however, they continued to have higher lipid accumulation than normal PHHs (100% ± 35.9).

**Figure 2:**
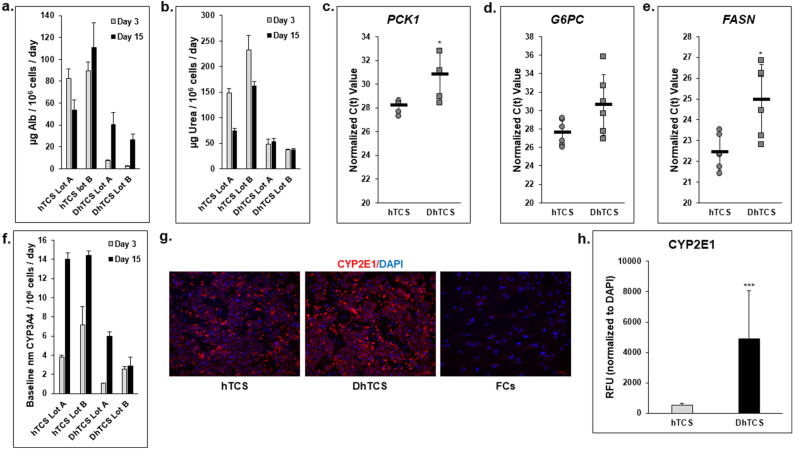
Determination of functional differences in hTCS and DhTCS PHHs. Representative levels of (**a**) albumin and (**b**) urea produced by hTCS and DhTCS PHHs on days 3 (gray bars) and 15 (black bars) (n = 3 replicates, n = 2 donors per condition). Gene expression represented by C(t) values of (**c**) *PCK1*, (**d**) *G6PC*, and (**e**) *FASN* on day 15 from hTCS and DhTCS PHHs (n ≥ 2 replicates, n ≥ 3 donors per condition) (Student’s t-test). (**f**) Representative baseline or uninduced CYP3A4 activity in hTCS and DhTCS PHHs on days 3 (gray bars) and 15 (black bars) (n = 3 replicates, n = 2 donors per condition). (**g**) Representative images of CYP2E1 (red) plus DAPI (blue) from hTCS and DhTCS PHHs and Feeder Cells (FC) on day 15. Magnification 10X. Scale bar = 100 µm. (**h**) Quantitation of CYP2E1 expression normalized to DAPI expression in hTCS PHHs (gray bars) and DhTCS PHHs (black bars) on day 15 (n = 5 images per donor, n ≥ 2 donors per condition) (Student’s *t* test). For (**a**), (**b**), and (**f**), values were normalized to the number of attached PHHs for each lot. *p ≤ 0.05, ***p ≤ 0.001 to hTCS PHHs. Error bars represent standard deviation.

The functionality of each group of PHHs was measured and normalized to attached PHHs in the hTCS and DhTCS (Fig. 2). Secretion of albumin (Fig. 2a) and urea (Fig. 2b) were greater in normal PHH lots compared to diseased lots on days 3 and 15. Expression of two genes related to production of enzymes that are critical to gluconeogenesis, *PCK1* (Fig. 2c) and *G6PC* (Fig. 2d)*,* were examined on day 15^21^. Diseased PHHs had lower gene expression of both these genes compared to normal PHHs. The average C(t) value for *PCK1* in diseased PHHs was 30.8 ± 2.1 compared to the value in normal PHHs of 28.2 ± 0.7. C(t) values for *G6PC* were higher in diseased versus normal PHHs (30.6 ± 3.3 vs 27.6 ± 1.4). Gene expression of *FASN* was measured, and significantly higher C(t) values were seen in diseased PHHs than in normal PHHs (25.0 ± 1.7 vs 22.4 ± 0.8) (Fig. 2e).

CYP3A4 activity, normalized to the number of attached PHHs, was lower in diseased PHHs when compared to normal PHHs on days 3 and 15 (Fig. 2f). By contrast, CYP2E1 immuno-reactive staining was more intense in diseased PHHs compared to normal PHHs on day 15, while FCs alone exhibited little to no staining (Fig. 2g). Image analysis showed there was significantly higher CYP2E1 protein expression in diseased PHHs (4893 ± 3187 RFUs) versus normal PHHs (542 ± 133 RFUs) (Fig. 2h).

### Diseased PHHs and inflammation

**Figure 3:**
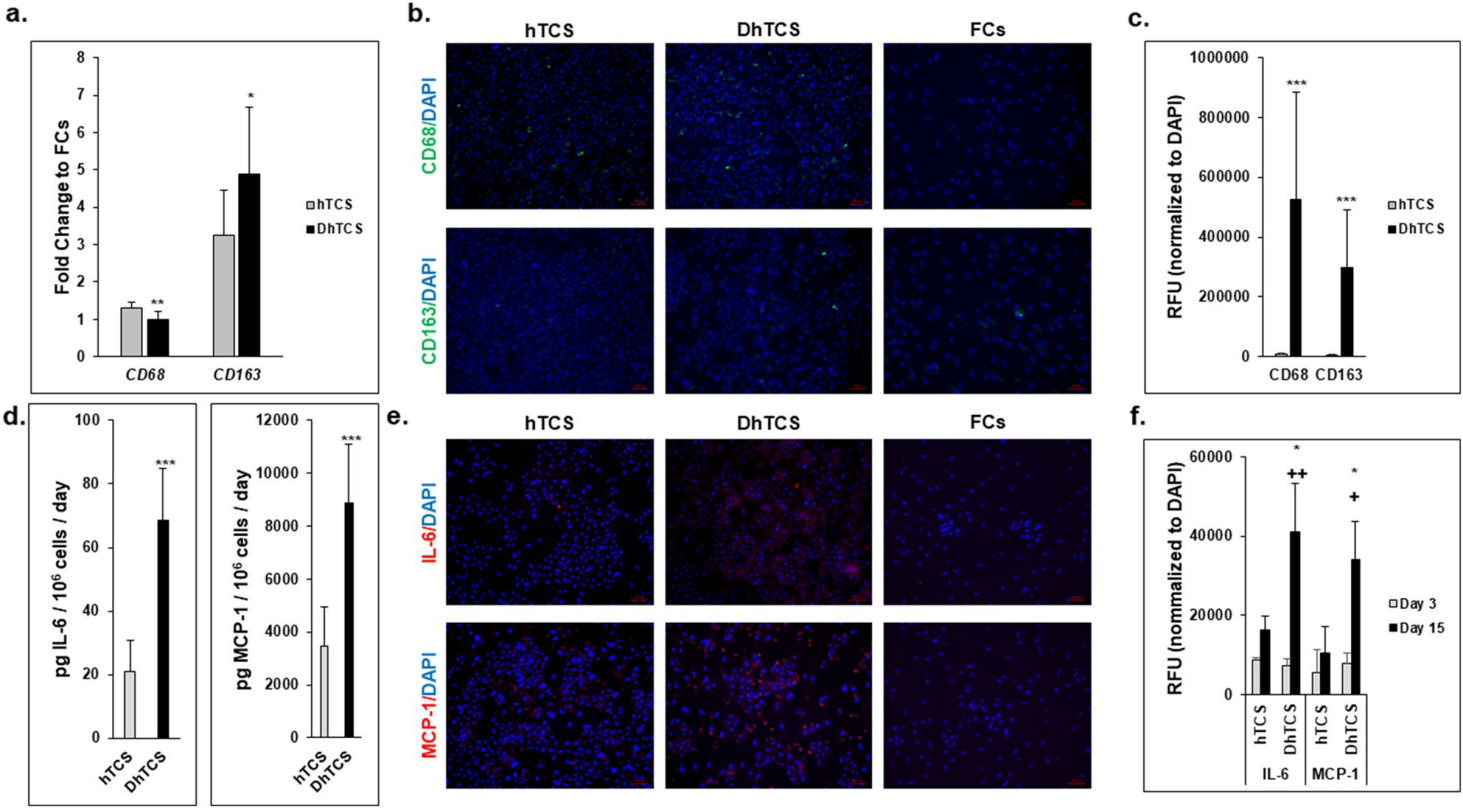
Determination of macrophage and cytokine expression in normal and diseased PHHs in the hTCS and DhTCS. (**a**) Gene expression represented as fold change to Feeder Cells (FCs) of *CD68* and *CD163* in hTCS (gray bars) and DhTCS (black bars) on day 15 (n = 2 replicates, n = 3 donors per condition). (**b**) Expression of macrophage markers CD68 (green, top row) and CD163 (green, bottom row) plus DAPI (blue) for nuclear stain in hTCS (left column), DhTCS (middle column), and Feeder Cells (FCs) (right column) on day 15. Magnification 10X. Scale bar = 100 µm. (**c**) Quantitation of CD68 and CD163 expression in hTCS (gray bars) and DhTCS (black bars) on day 15 (n = 5 images per donor, n = 3 donors per condition). Values were normalized to DAPI expression. (**d**) Levels of IL-6 and MCP-1 were measured in hTCS (gray bars) and DhTCS (black bars) on day 15. Values were normalized to the number of attached PHHs for each lot tested in each system (n = 2 replicates, n = 2 donors per condition). (**e**) Representative images of nuclear stain DAPI (blue) and either IL-6 (red, top row) or MCP-1 (red, bottom row) in hTCS (left column), DhTCS (middle column), and FCs (right column) on day 15. Magnification 10X. Scale bar = 100 µm. (**f**) Quantitation of IL-6 and MCP-1 expression normalized to DAPI expression in hTCS and DhTCS on days 3 (gray bars) and 15 (black bars) (n = 2 images per donor, n = 2 donors per condition). Student’s *t* test for (**a**), (**c**), (**d**). One-way ANOVA with Tukey for (**f**). *p ≤ 0.05, ** p ≤ 0.01, ***p ≤ 0.001 to hTCS on day 15; ^+^*p* ≤ 0.05, ^++^*p* ≤ 0.01 to DhTCS on day 3. Error bars represent standard deviation.

The link between macrophage and inflammatory markers was examined in diseased and normal PHHs (Fig. 3). The presence of resident macrophages (a normal low-frequency contaminant of PHH preparations) in the hTCS and DhTCS was determined by measuring gene expression of *CD68* and *CD163* on day 15 (Fig. 3a). A significant fold change difference was seen in *CD68* expression between the two systems (diseased fold-change 1.0 ± 0.2 vs normal fold-change 1.3 ± 0.2). *CD163* gene expression was significantly higher in the DhTCS (4.9 ± 1.8) compared to the hTCS (3.3 ± 1.2). Immunostaining for CD68 and CD163 was performed in the hTCS, DhTCS, and FCs on day 15 (Fig. 3b). While FCs alone exhibited little to no positive staining, quantitation of CD68 + and CD163 + cells revealed a significantly higher fraction of macrophages in the DhTCS (CD68: 524,578 ± 358,720 and CD163: 299,177 ± 192,429) compared to hTCS (CD68: 7039 ± 3788 and CD163: 5808 ± 2264) on day 15 (Fig. 3c).

Gene and protein expression of the pro-inflammatory cytokines IL-6 and MCP-1 were determined on day 15. *IL-6* gene expression was significantly higher in the DhTCS compared to the hTCS (Supplementary Fig. 3). There was ≥ threefold-change in *IL-6* gene expression in the DhTCS compared to the hTCS relative to FCs resulting in a significant difference between the two systems (3.6 ± 2.7 vs 1.0 ± 0.5). No significant difference was seen in *MCP-1* gene expression between the two systems. The fold-change for *MCP-1* expression was under twofold for DhTCS compared to the hTCS relative to FCs (0.7 ± 0.2 vs 0.5 ± 0.3).

When IL-6 and MCP-1 amounts were measured, the DhTCS secreted significantly higher levels of IL-6 (68.5 ± 16.4 *vs* 21.1 ± 9.7 pg/10^6^ cells/day) and MCP-1 (8888.8 ± 2195.1 vs 3482.2 ± 1484.5 pg/10^6^ cells/day) on day 15 (Fig. 3d). The DhTCS and hTCS were stained for expression of IL-6 and MCP-1 on days 3 (Supplementary Fig. 3) and 15 (Fig. 3e). Higher intensity immunostaining of both were seen in the DhTCS compared to the hTCS on only day 15, while FCs alone exhibited little to no staining. Quantification of the fluorescent signal determined levels of IL-6 and MCP-1 expression (Fig. 3f). A significantly higher expression level in the DhTCS over the hTCS was determined for IL-6 (41,060 ± 12,259 vs 16,435 ± 3325 RFUs) and MCP-1 (33,960 ± 9650 vs 10,645 ± 6652 RFUs).

**Figure 4:**
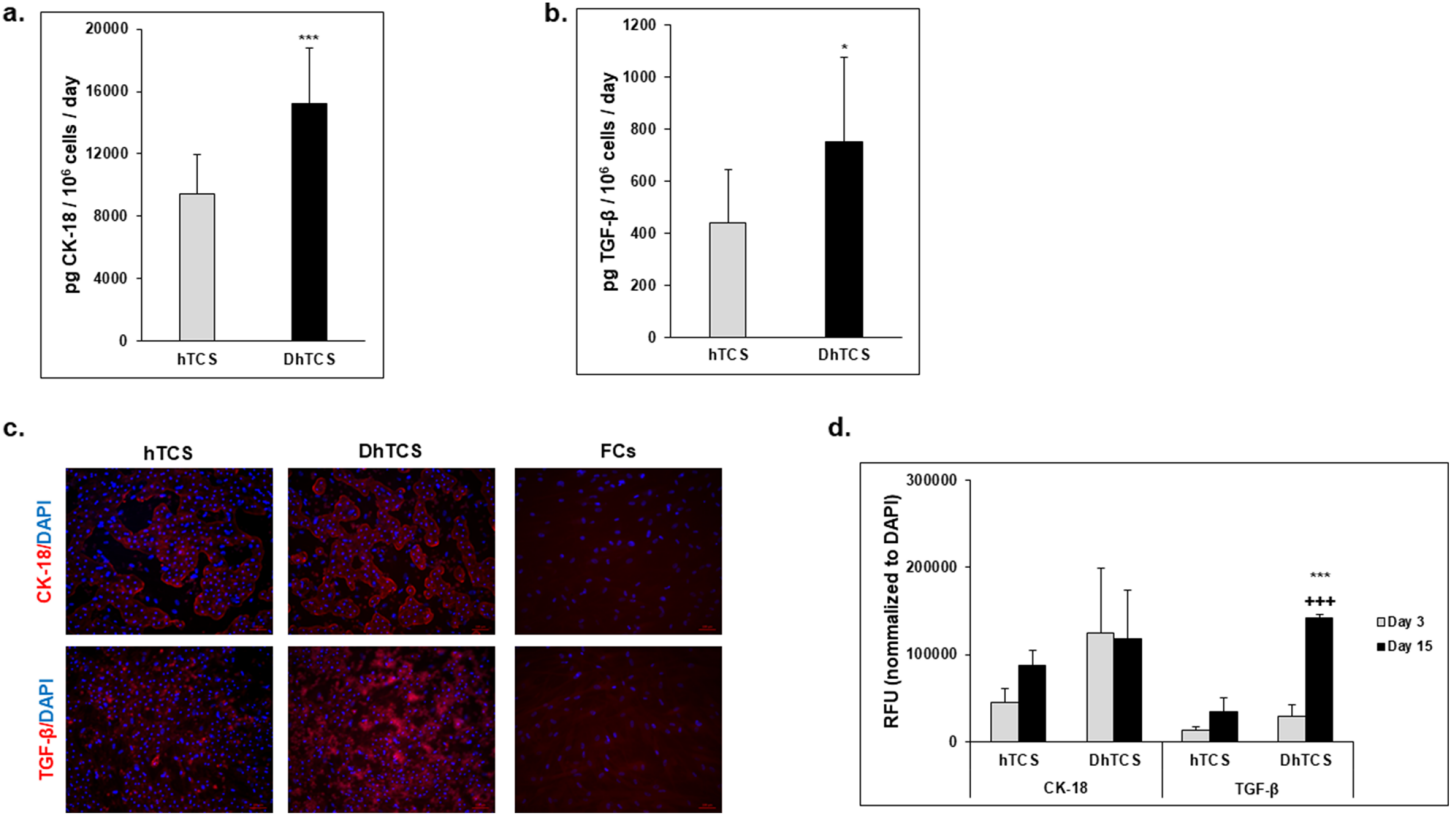
Diseased PHHs show expression of fibrotic markers. Levels of (**a**) CK-18 and (**b**) TGF-β were measured in hTCS (gray bars) compared to DhTCS (black bars) on day 15. (n = 3 replicates, n = 2 donors per condition) (Student’s *t* test). (**c**) Representative images of nuclear stain DAPI (blue) and either CK-18 (red, top row) or TGF-β (red, bottom row) in hTCS (left column), DhTCS (middle column), and Feeder Cells (FCs) (right column) on day 15. Magnification 10X. Scale bar = 100 µm. (**d**) Quantitation of CK-18 and TGF-β normalized to DAPI expression in hTCS and DhTCS on days 3 (gray bars) and 15 (black bars) (n = 2 images per donor, n = 2 donors per condition) (One-way ANOVA with Tukey). For (**a**) and (**b**), values were normalized to the number of attached PHHs for each lot tested in each system. *p ≤ 0.05, ***p ≤ 0.001 to hTCS PHHs on day 15; ^+++^*p* ≤ 0.001 to DhTCS on day 3. Error bars represent standard deviation.

The expression and production of fibrotic markers, CK-18 and TGF-β, were observed in the DhTCS and hTCS (Fig. 4). *CK-18* and *TGF-β* gene expression was determined relative to FCs on day 15 (Supplementary Fig. 3). There was no significant difference between the DhTCS (2.9 ± 0.3) and hTCS (3.2 ± 0.5) for *CK-18* gene expression. *TGF-β* gene expression was significantly higher expressed in the DhTCS (3.0 ± 1.4) compared to the hTCS (0.5 ± 0.2). The cells in the DhTCS secreted significantly higher levels of CK-18 (15,224 ± 3569 vs 9462 ± 2461 pg/10^6^ cells/ day) and TGF-β (753.4 ± 322.4 vs 439.5 ± 205.4 pg/10^6^ cells/day) compared to cells in the hTCS (Fig. 4a,b). Higher intensity staining was seen for CK-18 and TGF-β in the DhTCS on days 3 (Supplementary Fig. 3) and 15 (Fig. 4c) compared to the hTCS. Quantification of CK-18 determined there was no change in staining between the DhTCS and hTCS from day 3 (124,629 ± 74,838 vs 45,698 ± 15,386 RFUs) to 15 (117.532 ± 56.475 vs 87.264 ± 17.596 RFUs) (Fig. 4d). TGF-β immunostaining was significantly higher in the DhTCS compared to the hTCS on day 15 (141,968 ± 4079 vs 34,005 ± 16,761 RFUs). There was a significant increase in immunostaining in cells from the DhTCS from day 3 (29,014 ± 13,721 RFUs) to 15 but not in cells from the hTCS from day 3 (13,383 ± 4132) to 15. By contrast, FCs alone exhibited little to no immunostaining.

### Establishing a lipogenesis model

**Figure 5:**
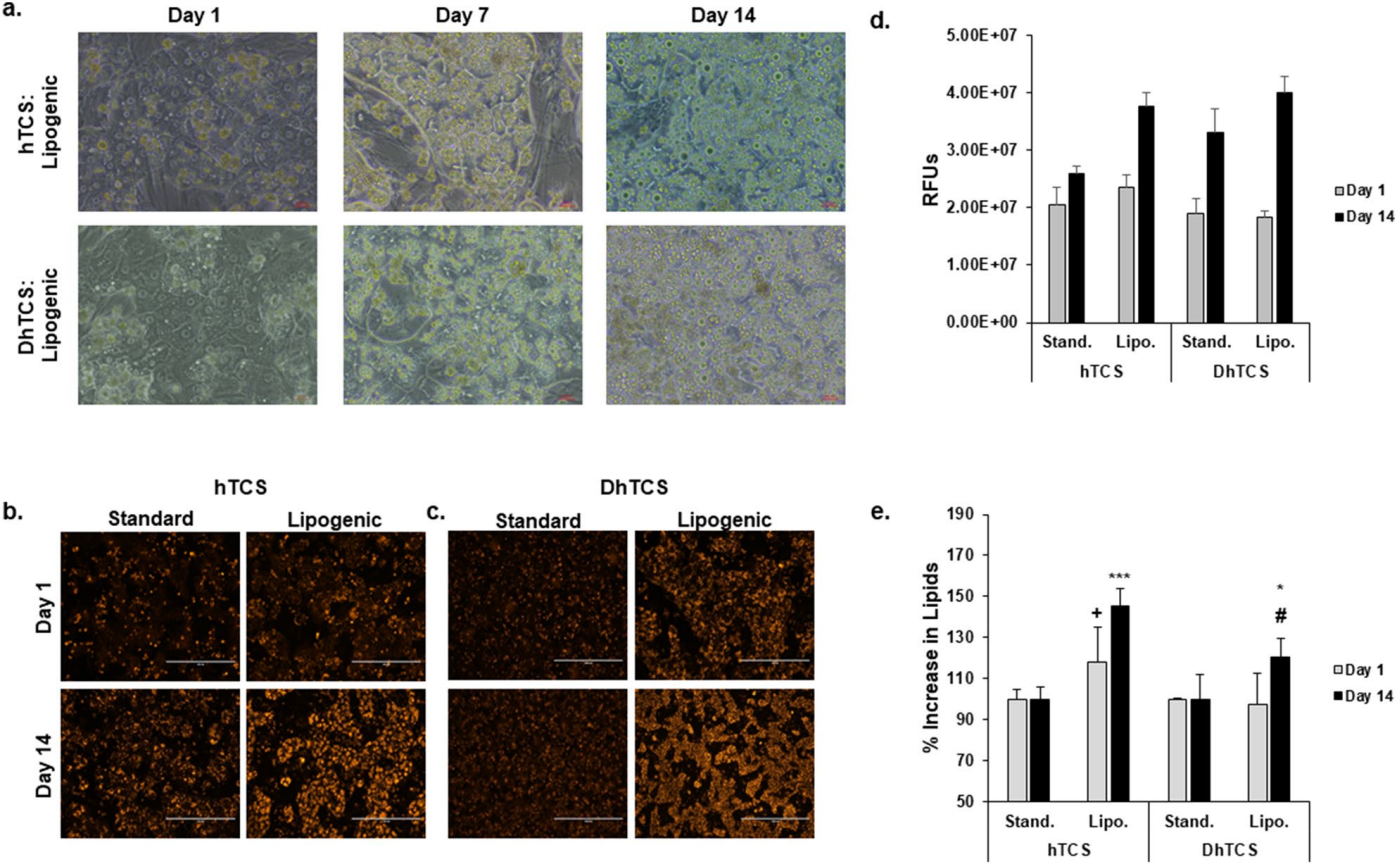
Normal and diseased PHHs cultured in lipogenic medium increases lipid. expression. (**a**) Representative images of normal PHHs (top row) and diseased PHHs (bottom row) cultured in lipogenic (Lipo.) medium (oleic acid: 320 µM and high glucose: 25 mM) in the hTCS and DhTCS on days 1, 7, and 14. Magnification 20X. Scale bar = 100 µm. Representative images of Nile Red staining of (**b**) standard (Stand.) and lipo. medium cultured normal PHHs in the hTCS and (**c**) stand. and lipo. medium cultured diseased PHHs in the DhTCS on days 1 and 14. Magnification 10X. Scale bar = 400 µm. (**d**) Quantification of Nile Red Staining in Relative fluorescent units (RFUs) of stand. and lipo. medium cultured normal PHHs in the hTCS and stand. and lipo. medium cultured diseased PHHs in the DhTCS on days 1 (gray bars) and 14 (black bars) (n ≥ 4 images per condition, n = 2 donors for hTCS; n ≥ 3 images per condition, n = 1 donor for DhTCS). (**e**) Percent increase in lipid expression measured by Nile Red staining of normal and diseased PHHs cultured in stand. and lipo. medium on days 1 (gray bars) and 14 (black bars) in the hTCS and DhTCS (n ≥ 4 images per condition, n = 2 donors for hTCS; n ≥ 3 images per condition, n = 1 donor for DhTCS). *p ≤ 0.05, ***p ≤ 0.001 to untreated PHHs on day 14. + *p* ≤ 0.05 to untreated PHHs on day 1. # *p* ≤ 0.05 to normal lipogenic treated PHHs on day 14. One-way ANOVA with Tukey for stats. Error bars represent standard deviation.

An important feature of the NAFLD-related hepatocellular phenotype is steatosis. To induce lipid production, PHHs in the hTCS and DhTCS were cultured with a medium that included high glucose (25 mM) and the FFA oleic acid (320 µM) (lipo medium) (Fig. 5). Normal and diseased PHHs were morphologically similar in cuboidal cell shape and forming multicellular clusters and exhibited an increase in lipid accumulation throughout the 14-day culture period when cultured in lipo medium (Fig. 5a), which was confirmed by a corresponding increase in Nile red staining over the two-week period (Fig. 5b,c). Quantification of the Nile Red staining showed higher values on day 1 in normal PHHs cultured in the lipo versus standard medium; however, similar values were seen in the DhTCS PHHs for both medias (Fig. 5d). Nile Red staining increased in the normal PHHs in the hTCS from day 1 to 14 in the lipo medium group. Both standard and lipo medium conditions increased Nile Red staining in the diseased PHHs in DhTCS between day 1 and 14. Normalization showed a significant increase in lipid production between standard and lipo medium cultured PHHs for normal (100% ± 5.6 vs 145.3% ± 8.5) and diseased PHHs (100% ± 11.8 *vs* 120.6% ± 9.1) in the hTCS and DhTCS on day 14 (Fig. 5e). A significant increase in lipid accumulation occurred in lipo medium cultured PHHs on day 1 from normal (118.2% ± 16.8) donors but not diseased donors (97.2% ± 15.4).

### Functionality of PHHs cultured in lipogenic medium

**Figure 6:**
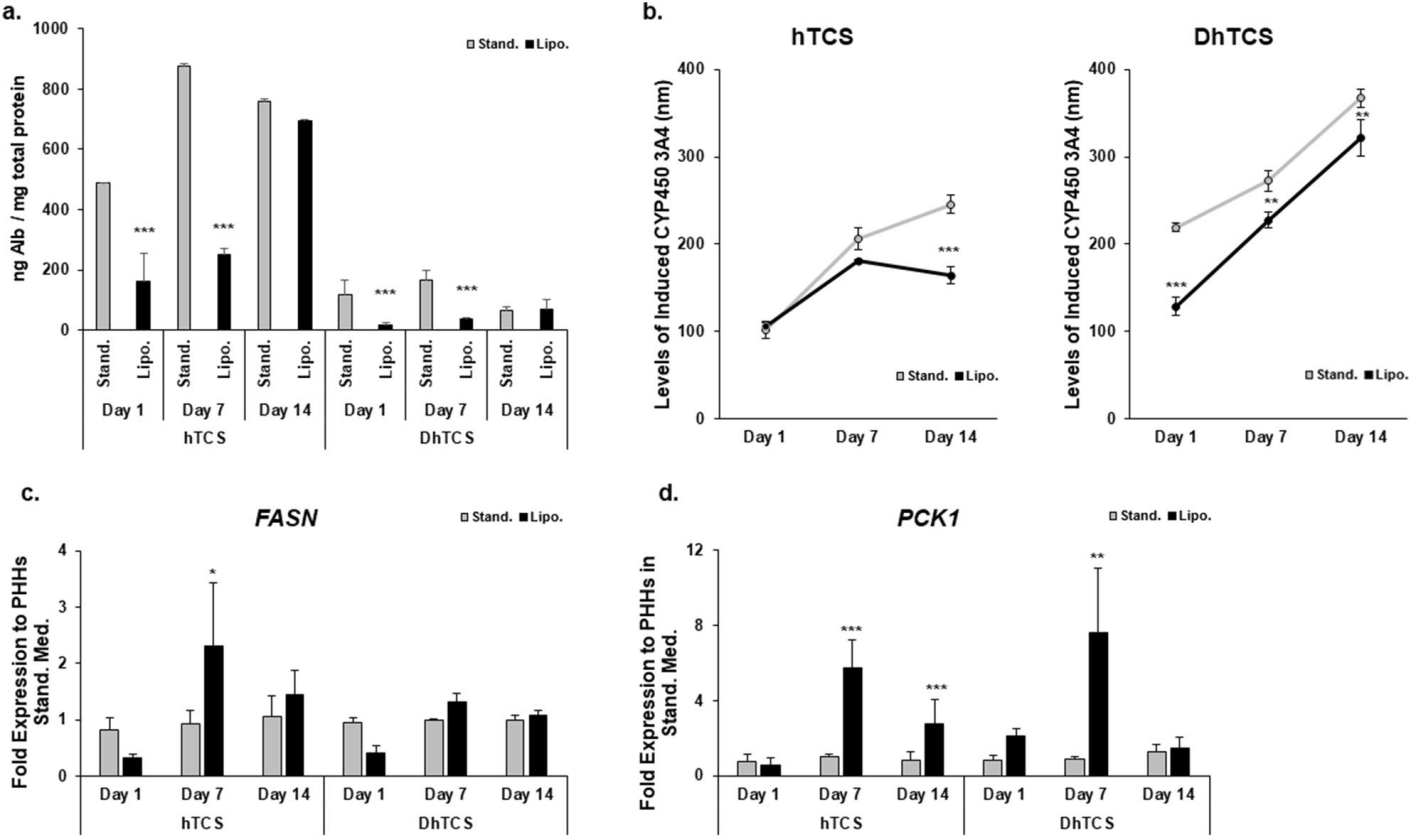
Culturing of PHHs in lipogenic medium decreases functionality in normal and diseased PHHs. (**a**) Levels of albumin measured on days 1, 7, and 14 in normal and diseased PHHs in the hTCS and DhTCS cultured in standard medium (Stand., gray bars) and lipogenic medium (Lipo., black bars) (oleic acid: 320 µM and high glucose: 25 mM). Values were normalized to total protein concentration for each sample in each condition. (n = 3 replicates per condition, n = 2 donors for hTCS; n = 1 donor for DhTCS) (One-way ANOVA with Tukey). (**b**) Induced CYP3A4 activity measured in normal and diseased PHHs in the hTCS and DhTCS on days 1, 7, and 14 cultured in stand. culture medium (gray line) and lipo. medium (black line) PHHs. (n ≥ 3 replicates per condition, n = 2 donors for hTCS; n = 1 donor for DhTCS) (One-way ANOVA with Tukey). Gene expression of (**c**) *FASN* and (**d**) *PCK1* in normal and diseased PHHs in the hTCS and DhTCS on days 1, 7, and 14 cultured in stand. medium (gray bars) and lipo. medium (black bars). (n ≥ 4 replicates per condition, n = 2 donors for hTCS; n = 1 donor for DhTCS) (One-way ANOVA with Tukey). *p ≤ 0.05, **p ≤ 0.01, ***p ≤ 0.001 to standard cultured PHHs on specified day. Error bars represent standard deviation.

Albumin secretion and CYP3A4 activity were used as representative functions to determine perturbations in PHHs cultured in the lipo medium in the hTCS and DhTCS (Fig. 6). Overall, diseased PHHs secreted less albumin compared to normal PHHs (Fig. 6a). The albumin levels decreased on days 1 and 7 in normal and diseased PHHs cultured in the lipo medium. There was a rise in albumin levels over time in the lipo cultured normal and diseased PHHs resulting in almost equivalent levels by day 14 between both medium groups in the hTCS and DhTCS.

Differences in CYP3A4 activity were measured between standard medium and lipo medium cultured groups in normal and diseased PHHs in the hTCS and DhTCS (Fig. 6b). No difference in induced CYP3A4 activity was seen on day 1 between standard medium (101.7 ± 10.2 nm) and lipo medium cultured (105.3 ± 4.3 nm) normal PHHs. Differences in induced CYP activity were seen on days 7 (181.4 ± 0.8 nm vs 205.7 ± 12.6 nm) and 14 (164.5 ± 9.9 nm vs 245.5 ± 10.5 nm) with day 14 being significantly higher in the standard medium cultured samples. Lower induced CYP activity was seen in diseased PHHs cultured in lipo medium compared to the diseased PHHs cultured in standard medium at day 1 (128.4 ± 10.5 nm vs 219.2 ± 5.3 nm). Lipo medium cultured diseased PHHs continued to have lower activity on days 7 (227.3 ± 9.2 nm vs 272.2 ± 12.1 nm) and 14 (321.8 ± 20.6 nm vs 367.1 ± 10.2 nm).

Gene expression of *FASN* (Fig. 6c) and *PCK1* (Fig. 6d) was determined in normal and diseased PHHs maintained in the standard *vs* lipo medium. In normal and diseased PHHs, *FASN* gene expression was lower on day 1 in lipo medium cultured PHHs compared to PHHs cultured in standard medium. A significant increase in *FASN* gene expression was measured on day 7 in normal PHHs cultured in lipo medium (2.3 ± 1.2) compared to standard medium cultured PHHs (0.9 ± 0.2), but no significant increase was measured on day 14 (1.5 ± 0.4 vs 1.1 ± 0.4). No significant differences were seen in the diseased PHHs cultured in standard medium or lipo medium on days 7 and 14. *PCK1* expression was significantly higher on day 7 in the normal PHHs cultured in lipo medium (5.8 ± 1.5 vs 1.0 ± 0.1) and the diseased PHHs cultured in the lipo medium (7.6 ± 3.5 vs 0.9 ± 0.1) compared to the standard medium cultured PHHs for each group. Significantly elevated expression continued in the lipo medium cultured normal PHHs on day 14 (2.8 ± 1.3 vs 0.8 ± 0.4) but not in the lipo medium cultured diseased PHHs (1.5 ± 0.6 vs 1.3 ± 0.4).

### Lipogenic medium plus OCA treatment in the hTCS

**Figure 7:**
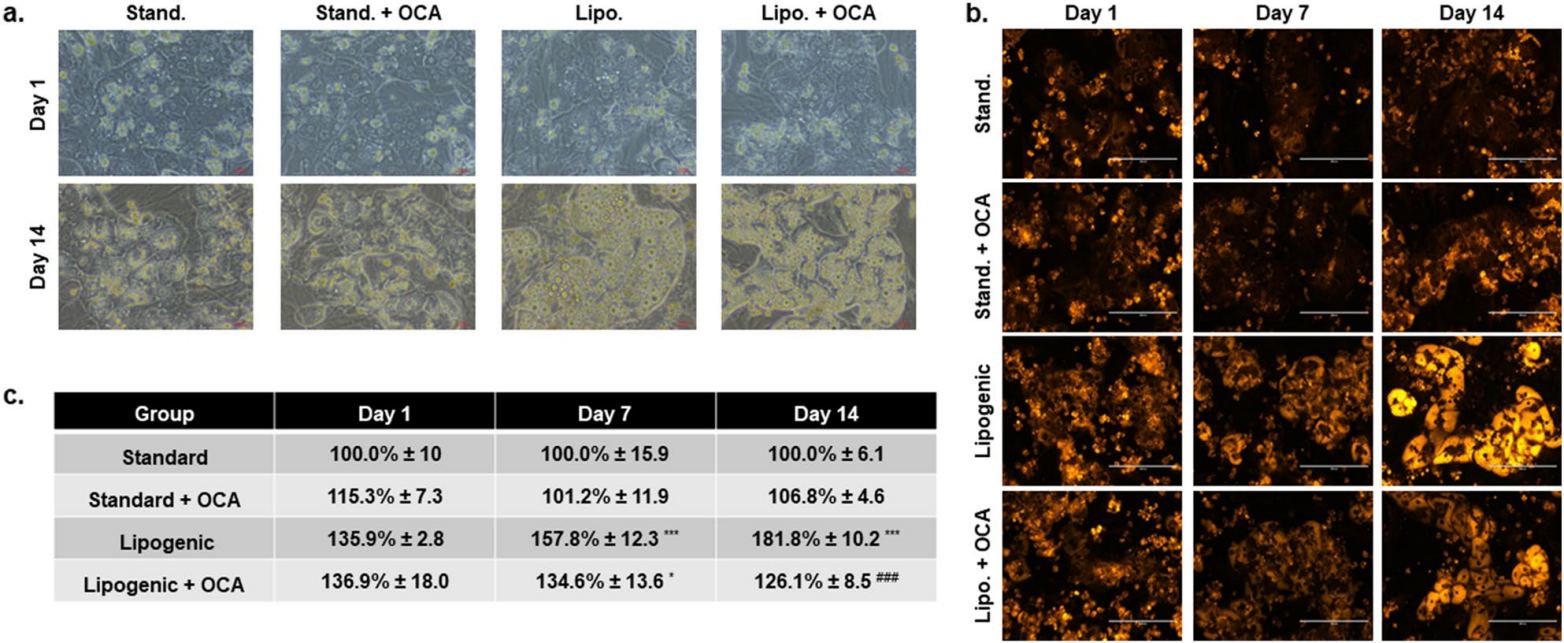
Adding Obeticholic acid to lipogenic culture medium decreases lipid expression. (**a**) Representative phase contrast images of normal PHHs cultured in standard medium (Stand.), stand. medium with Obeticholic acid (OCA, 0.5 µM), lipogenic (Lipo.) medium (oleic acid : 320 µM and high glucose : 25 mM), and lipo. medium plus OCA (0.5 µM) on days 1 and 14 in the hTCS. Magnification 20X. Scale bar = 100 µm. (**b**) Nile red staining images of normal PHHs cultured in stand. medium, stand. medium with OCA, lipo. medium, and lipo. medium plus OCA on days 1, 7, and 14 in the hTCS. Magnification 20X. Scale bar = 200 µm. (**c**) Quantitation of lipid expression measured by Nile Red staining of PHHs cultured in stand. medium, stand. medium with OCA, lipo. medium, and lipo. medium plus OCA on days 1, 7, and 14 in the hTCS. (n ≥ 2 images per condition, n = 2 donors). *p ≤ 0.05, ***p ≤ 0.001 to normal PHHs on specified day. ^###^*p* ≤ 0.001 to lipogenic treated PHHs on day 14. One-way ANOVA with Tukey. Average and standard deviations are shown.

PHHs prepared from healthy liver tissues were seeded in the hTCS and cultured in standard culture or lipo medium, with or without OCA treatment, a potent agonist of the bile acid receptor FXR (Fig. 7). Representative images demonstrate lipid accumulation when PHHs were cultured in the lipo medium with or without OCA treatment on days 1 and 14 (Fig. 7a). When OCA was added with the lipo medium, less lipid accumulation was apparent on day 14 determined by Nile red staining (Fig. 7b). Lipid accumulation increased on days 1 (135.9% ± 2.8), 7 (157.8% ± 12.3), and 14 (181.8% ± 10.2) in the PHHs cultured in lipo medium (Fig. 7c). Lipid accumulation was decreased on days 7 (134.6% ± 13.6) and 14 (126.1% ± 8.5) in the lipo medium plus OCA cultured PHHs compared to the lipo medium only cultured PHHs with day 14 having significantly less lipid accumulation.

**Figure 8:**
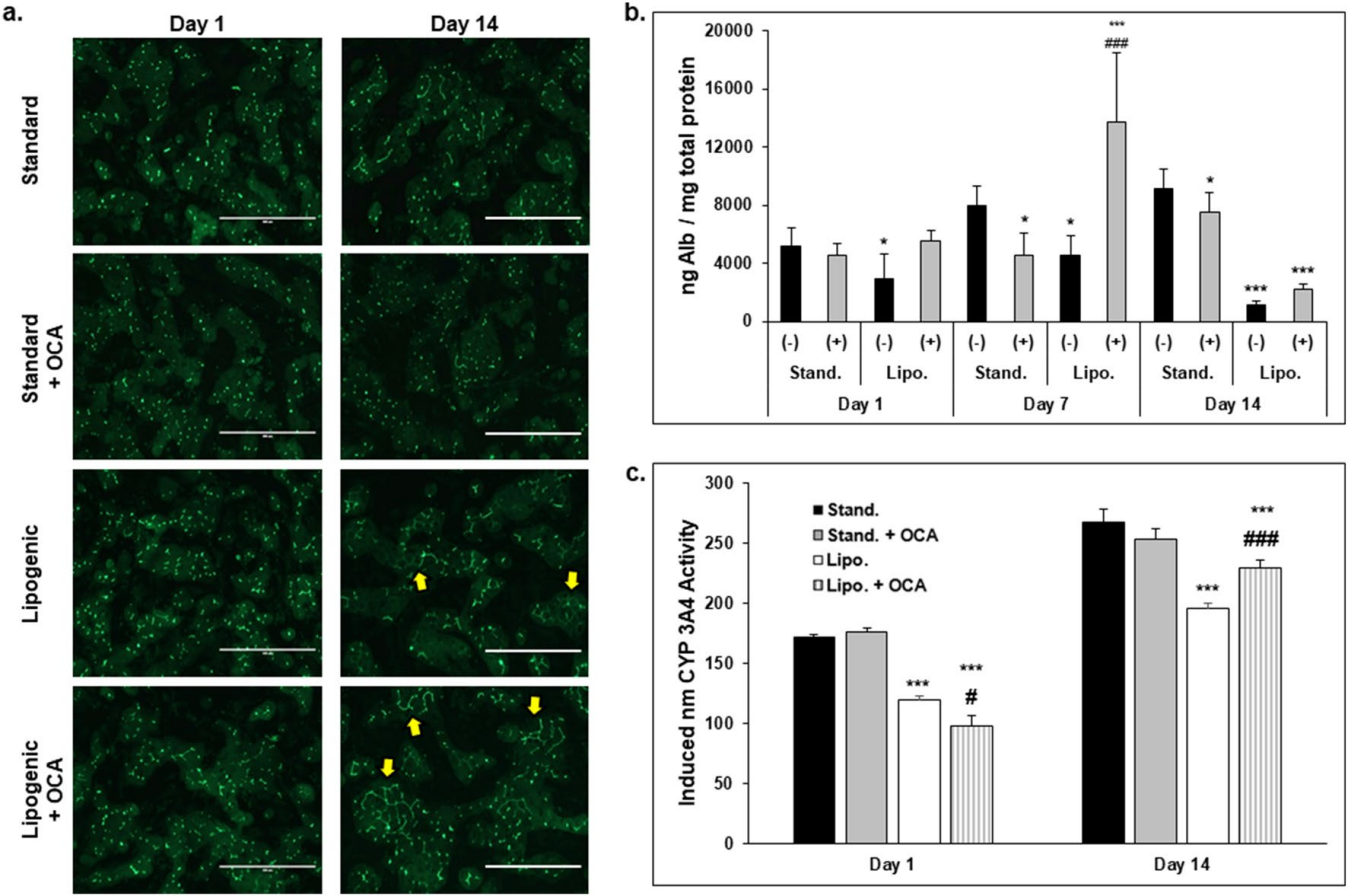
Obeticholic acid increases functionality in PHHs cultured in lipogenic medium. (**a**) Bile canaliculi staining in normal PHHs cultured in standard (Stand.) medium, stand. medium with Obeticholic acid (OCA, 0.5 µM), lipogenic (Lipo.) medium (oleic acid : 320 µM and high glucose: 25 mM), and lipo. medium plus OCA (0.5 µM) on days 1 and 14 in the hTCS. Magnification 10X. Scale bar = 400 µm. Arrows indicate extensive bile canalicular networking. (n ≥ 3 images per condition, n = 1 donor). (**b**) Levels of albumin produced by stand. and lipo. medium cultured normal PHHs without (–, black bars) or with (+ , grey bars) the addition of OCA on days 1, 7, and 14. Values were normalized to total protein concentration for each sample in each condition (n = 3 replicates per condition, n = 2 donors) (One-way ANOVA with Tukey). (**c**) Induced CYP3A4 activity measured in normal PHHs cultured in stand. medium (black bars), stand. medium with OCA (gray bars), lipo. medium (white bars), and lipo. medium plus OCA (striped bars) on days 1 and 14 (n = 3 replicates per condition, n = 2 donors) (One-way ANOVA with Tukey). *p ≤ 0.05, ***p ≤ 0.001 to normal PHHs on specified day. ^*#*^*p* ≤ 0.05, ^*###*^*p* ≤ 0.001 to lipogenic cultured PHHs on specified day. Error bars represent standard deviation.

Because OCA is used to treat primary biliary cholangitis, its effect on the structure and function of bile canaliculi in PHHs cultured in the hTCS was evaluated (Fig. 8). CDFDA staining showed similar bile canaliculi formation in normal PHHs maintained in standard medium in the presence or absence of OCA on days 1 and 14 (Fig. 8a). By contrast, normal PHHs cultured in the lipo medium with or without OCA treatment appeared to have altered bile canalicular network formation with more extensive branching compared to untreated PHHs.

Albumin secretion (Fig. 8b) and CYP3A4 activity (Fig. 8c) were measured in normal PHHs after culture in the lipo medium with or without OCA treatment. Albumin levels increased in the lipo medium plus OCA cultured PHHs compared to the lipo medium cultured PHHs on day 1, although not significantly. The greatest effect of OCA was measured on day 7 when the lipo medium plus OCA cultured PHHs had significantly higher albumin secretion (13,706 ± 4738 ng/mg total protein) compared to the lipo medium cultured PHHs (4527 ± 1393 ng/mg total protein) and standard medium cultured PHHs (7955 ± 1356 ng/mg total protein). By day 14, significantly lower albumin levels overall were observed in the lipo medium plus OCA cultured PHHs (2.219 ± 400 ng/mg total protein) compared to standard medium cultured PHHs (9142 ± 1324 ng/mg total protein), resulting in only slightly higher albumin levels compared to lipo medium cultured PHHs (1178 ± 252 ng/mg total protein).

When induced CYP3A4 activity was measured on day 1, lipo medium plus OCA cultured PHHs had the lowest induced CYP activity (97.5 ± 9.2 nm) compared to standard medium cultured PHHs (171.7 ± 2.1 nm) and lipo medium cultured PHHs (119.6 ± 3.5 nm). On day 14, lipo medium plus OCA cultured PHHs had significantly higher induced CYP3A4 activity (228.8 ± 6.7 nm) compared to lipo medium cultured PHHs (195.3 ± 4.4 nm) but lower induced activity compared to standard medium cultured PHHs (266.9 ± 11.3 nm). Overall, CYP3A4 activity was increased by OCA in the presence of increased FFA and high glucose.

## Discussion

A limitation with current in vitro models of NAFLD is the inability of diseased PHHs to attach and culture for extended periods of time^22^. Diseased PHHs were tested in the hTCS model system to determine the feasibility and their characteristics. Although there was a significant difference in attachment efficiency between diseased versus normal PHHs, diseased PHHs were able to attach and function for at least 2 weeks. Diseased PHHs showed a characteristic phenotype, including significant basal lipid accumulation. In terms of basic liver functions, the diseased DhTCS secreted less albumin and urea, and had lower baseline CYP3A4 activity when compared to normal PHHs after normalization. The decrease in albumin synthesis in the DhTCS is congruent with clinical observations of progressively impaired albumin levels in NAFLD patients^23^. Likewise, these findings are consistent with the impaired metabolic capacity in human liver microsomes and hepatocytes isolated from patients with advanced liver disease^24^.

Interestingly, the diseased PHHs exhibited several other advanced features of progressing NAFLD, including markers of inflammation. M2 macrophages were more prevalent in the DhTCS compared to the hTCS as detected by gene expression levels (*CD163*) and immunolocalization (CD163 and CD68) over the 14-day culture period. Both the chemokine MCP-1 and the pro-inflammatory cytokine IL-6 had higher protein expression in the DhTCS versus the hTCS. MCP-1 is a known recruiter of macrophages and has been shown to play a role in liver disease progression^25^. Serum levels of patients with NASH contained higher levels of IL-6 compared to NAFLD patients^26^. The increase in macrophage markers and elevated levels of pro-inflammatory cytokines in the DhTCS suggests that the model progressed beyond simple steatosis over the culture period.

Increased expression of TGF-β suggests that fibrogenic signaling cascades were activated in the DhTCS. The fibrosis scores from the donors were all ≤ 1; therefore, the severity of fibrosis would be expected to be minimal without further induction. In addition, CK-18 expression when measured with ICC remains constant in the DhTCS, suggesting the number of attached PHHs does not significantly change in either system over the culture period (Fig. 4d)^15^. However, TGF-β expression significantly increases in the DhTCS, indicating the diseased PHHs have a mild fibrotic background which can progress and lead to an inflammatory response (Fig. 4d). Ongoing studies are exploring the latter stages of the model and quantifying the relative expression and deposition of collagen I and collagen IV to the fibrogenic response in the DhTCS.

Gene expression related to fat and sugar utilization was altered in the diseased versus normal PHHs. In the DhTCS, expression of the *FASN* gene was lower in diseased PHHs compared to normal PHHs in the hTCS. Human livers have significantly higher expression of *FASN* in steatosis without significant inflammation as compared to control and NASH livers, suggesting that *FASN* expression is determined by the severity of disease^27^. The diseased PHHs maintained in the DhTCS may be in a “transitory phase” where the disease severity is progressing towards a fibrogenic state and a concomitant decrease in gene expression. A similar decrease in *PCK1* expression pattern was seen when transcriptome data sets were compared across progressive stages of fatty liver disease^28^. Gene expression from diseased PHHs cultured in the DhTCS was decreased for both *FASN* and *PCK1* compared to normal PHHs cultured in the hTCS, suggesting a moderately advanced diseased state. Further studies will assess whether glucose production is affected in both diseased and normal PHHs cultured in the DhTCS and hTCS.

The hTCS platform can support an “early stage” disease model through the addition of disease-relevant stimuli. A diseased state can be induced in both the hTCS and DhTCS by exposing the system to a combination of glucose and oleic acid. These two major stimuli, designated as lipogenic, were used to perturb normal and diseased PHHs. Notably, the glucose and insulin concentration in the standard culture medium can cause minor increases in lipid accumulation, which was seen in the hTCS. However, this increase was not significant and did not affect normal PHH functionality. However, significant differences in functionality between the diseased and normal PHHs maintained in lipogenic medium were seen in albumin secretion and induced CYP3A4 activity. It is interesting to note that the effects of the lipogenic medium on normal and diseased PHHs were not sustainable for albumin secretion. The albumin levels were almost equivalent between the two culture media in the hTCS and DhTCS on day 14, suggesting that the PHHs were able to restore function. Using higher FFA concentrations or a FFA mix may be required to determine if these effects are sustainable^11^.

FFA or high glucose treatment have been shown to cause similar decreases in CYP3A4 activity^29,30^. These results agree with the decreased activity that was measured in the advanced diseased PHHs without treatment in the DhTCS. Both types of PHHs showed an increase in lipid accumulation throughout the two-week culture period, although this increase was less significant in the treated diseased versus normal PHHs. These results imply that the diseased PHHs inherently have lower hepatic functions and exhibit a hyperlipidemic state, therefore additional lipogenic stimuli will have less effect compared to normal PHHs.

An increased sensitivity to lipogenic treatment was observed in the gene expression profiles of diseased PHHs. *PCK1* expression was increased in the lipogenic treated normal and diseased PHHs on day 7. Because *PCK1* plays a critical role in gluconeogenesis, the increase in gene expression suggests that changes in glucose production and insulin sensitivity may have occurred in the normal and diseased PHHs when treated with lipogenic media^31^. However, treated diseased PHHs appear less sensitive to this treatment perhaps due to their current state of disease. A similar result was seen in *FASN* expression with only treated normal PHHs showing increased expression. Normal PHHs cultured in a micropatterned co-culture exposed to hyperglycemic conditions for 3 weeks had increased *FASN* and *PCK1* expression on day 10 but decreased on day 18, similar to the pattern observed in the treated normal PHHs in the hTCS^30^. Based on these differences in response to treatment, normal PHHs cultured with lipogenic stimuli may be useful for “early stage” diseased modeling, where steatosis is important, while the use of diseased PHHs cultured in the DhTCS without stimuli may be applicable to a later stage of disease modeling.

Notably, the differences observed in attachment efficiency, gene expression profiles and other functional endpoints between hepatocytes isolated from donor tissues with lower versus higher NAS scores could be due to several cellular, positional, and pathological factors. It is not clear how much the attached cell populations are a representation of the overall hepatocyte populations from the respective tissues or how much they may represent a subpopulation that are being selected for during the preparation and seeding process. Marked differences in many facets of hepatocyte maturation, gene expression and disease biomarkers have been noted across the microarchitecture of the liver, e.g. periportal vs pericentral regions^22^. The cellular and molecular basis of these phenotypic differences observed in the hTCS have yet to be explored and may warrant further investigation.

Upon lipogenic treatment, OCA had an expected effect of decreasing lipogenesis in treated normal PHHs, a similar result found previously^32,33^, and affected the formation of the network of bile canaliculi. Since OCA is used to treat primary biliary cholangitis, a cholestatic disorder, enhancement of canalicular networks suggests the choleretic effects of OCA can be detected in the hTCS with treated normal PHHs. Hepatic functions, including albumin levels and CYP3A4 activity, were improved or restored upon OCA treatment in the PHHs cultured in the lipogenic medium. Likewise, an increase in CYP activity occurred when OCA was added to PHHs cultured with FFA medium compared to OCA untreated cultures^34^. Future studies are ongoing to investigate OCA-treated diseased PHHs in the DhTCS and evaluating its effect on hepatic functions and lipogenic responses, as well as mechanisms of bile acid mobilization and transport.

In conclusion, diseased PHHs cultured in the DhTCS were able to sustain distinct phenotypic features of fatty liver disease, including decreased hepatocyte functionality, altered lipogenic responses and pro-inflammatory cytokine production with elements of fibrogenesis. Diseased and normal PHHs responded to the lipogenic stimuli of high glucose and FFA by showing disease-associated features. OCA treatment, a drug known to reduce hepatic steatosis, modulated lipogenesis and functionality in lipogenic conditions. The DhTCS is a flexible and easy-to-use platform for investigation of liver disease at either an early “initiating” stage or a later “progressing” stage. Modulation of the standard hTCS model can be achieved by using disease-origin PHH (DhTCS) or introducing perturbations such as exposure to inducers of pathogenesis. Moreover, the longevity of the DhTCS system creates the potential for interventional strategies to be applied across the spectrum from prevention to treatment of advanced disease features.

### Supplementary Information

Supplementary Information.

## Data Availability

The datasets generated during and/or analyzed during the current study are available from the corresponding author on reasonable request.
