# Supplementary material for: Characterization of diseased primary human hepatocytes in an all-human cell-based triculture system
Source: Sci Rep. 2024 Mar 21;14:6772. doi: 10.1038/s41598-024-57463-7 (PMC10957907; doi:10.1038/s41598-024-57463-7)
Supplement: Supplementary file 1 — Supplementary Information. [file 41598_2024_57463_MOESM1_ESM.docx]

**Characterization of Diseased Primary Human Hepatocytes in an All-Human Cell-Based Triculture System**

Justin J. Odanga^1^, Sharon M. Anderson^1^, Erick K. Breathwaite^1^, Sharon C. Presnell^1^, Edward L. LeCluyse^2^, Jingsong Chen^1^, and Jessica R. Weaver^1*^

**
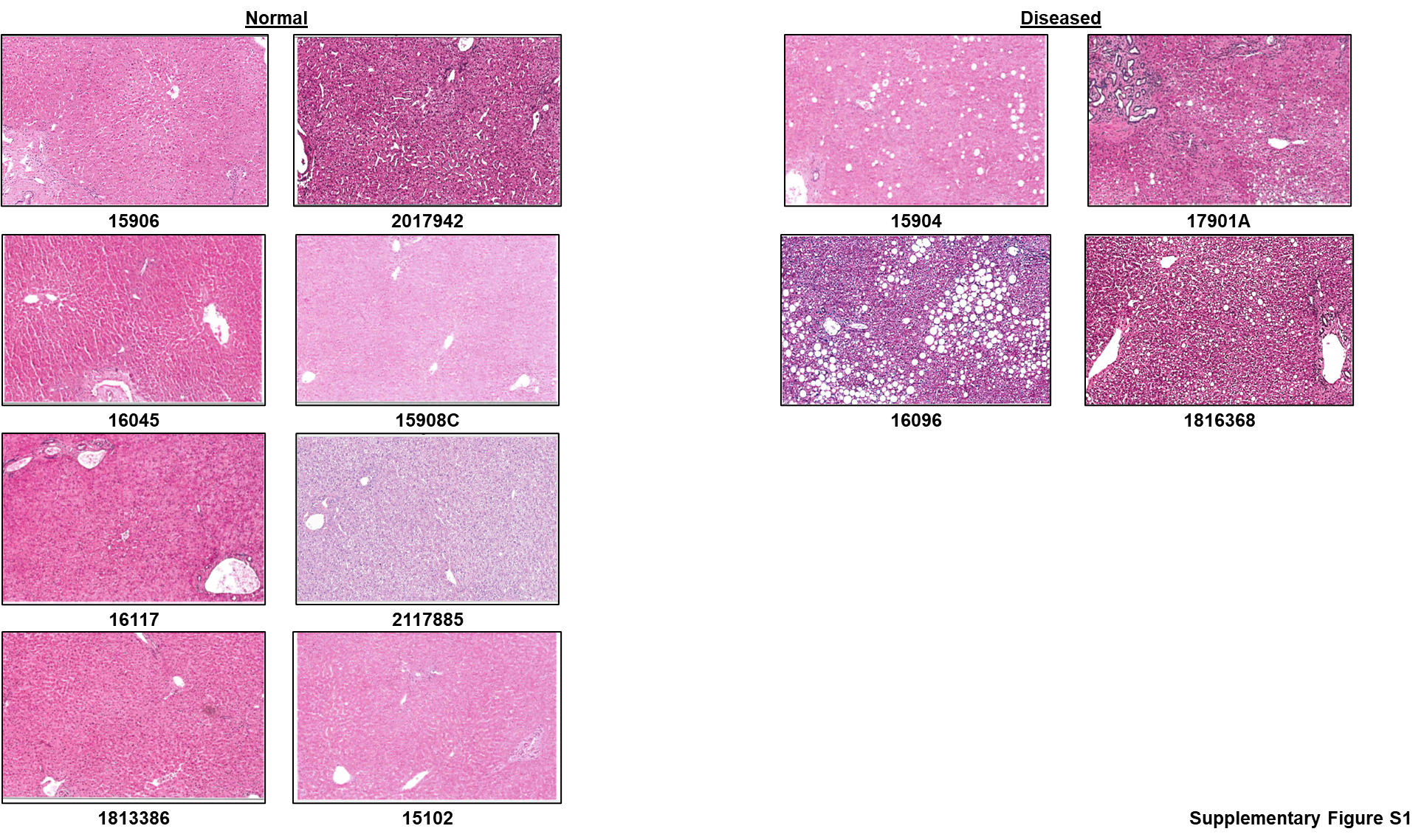
**

**Supplementary Figure S1.** H&E staining of liver tissue from normal donors and diseased donors listed in Supplementary Table 2.


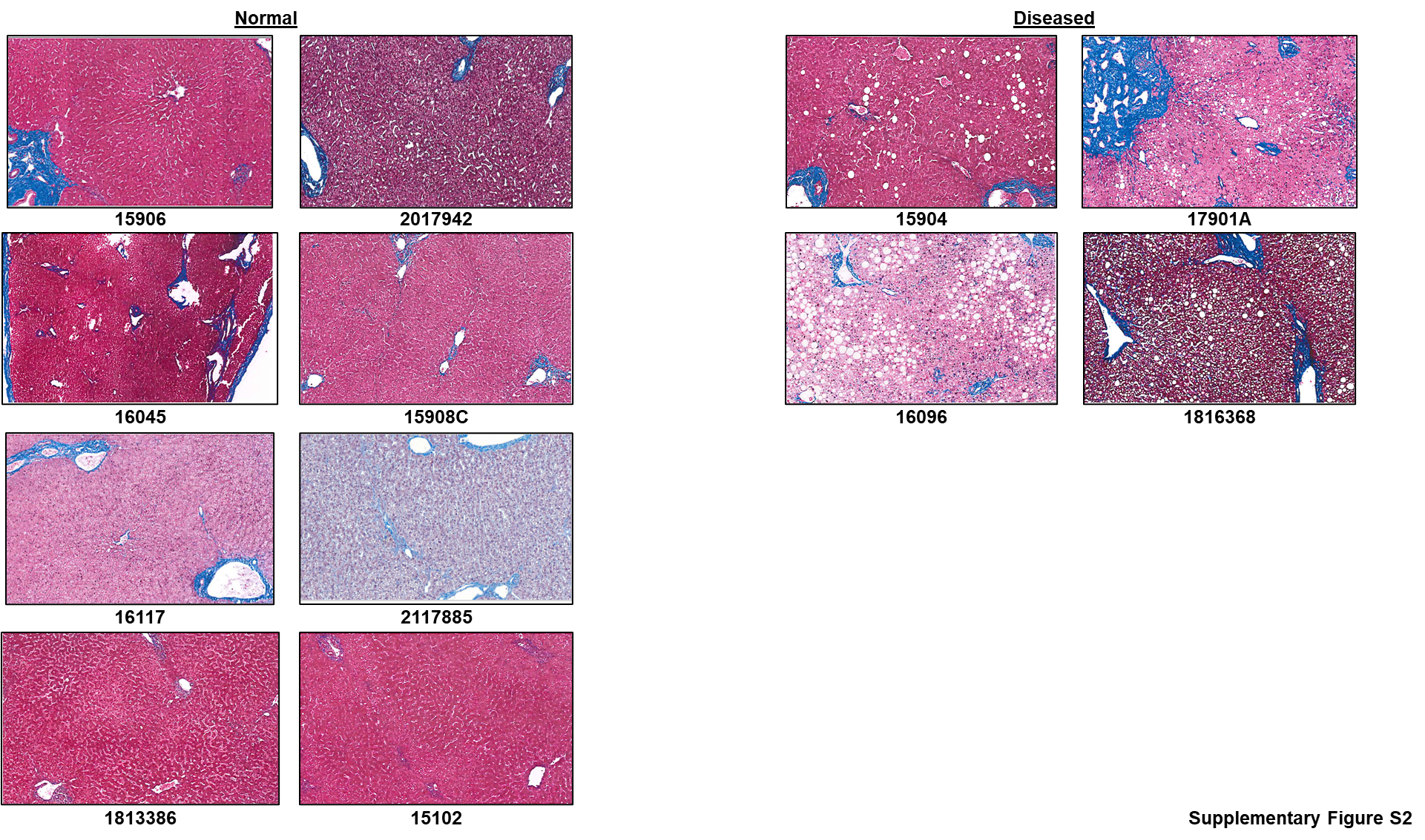


**Supplementary Figure S2.** Masson’s Trichrome staining of liver tissue from normal donors and diseased donors listed in

Supplementary Table 2.


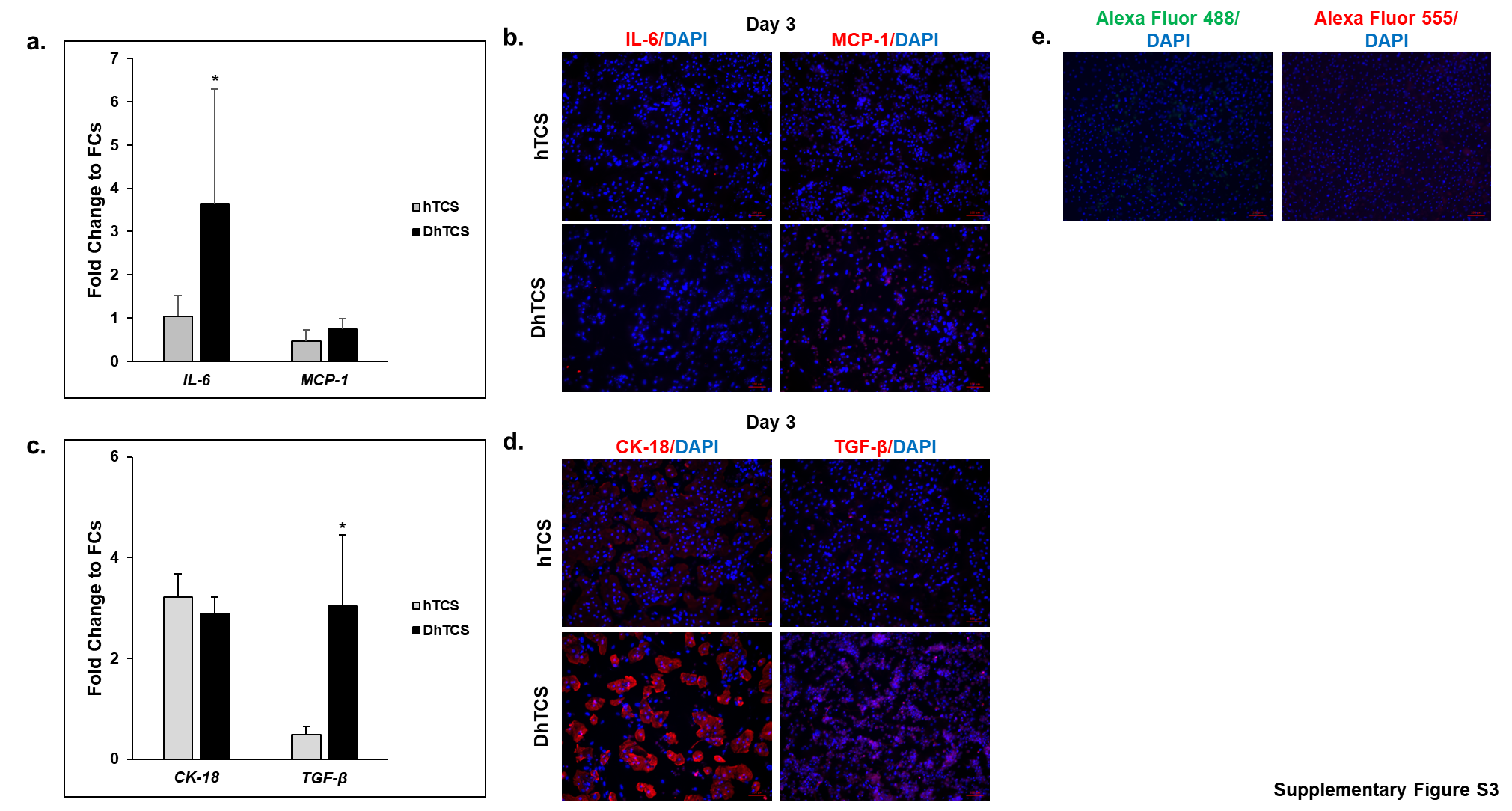


**Supplementary Figure S3**. (a) Gene expression represented as fold change to Feeder Cells (FCs) of *IL-6* and *MCP-1* in hTCS (gray bars) and DhTCS (black bars) PHHs on day 15 (n = 2 replicates, n ≥ 4 donors per condition) (Student’s t-test). (b) Representative images of DAPI (blue) nuclear stain and either IL-6 (red, left column) or MCP-1 (red, right column) in hTCS (top row) and DhTCS (bottom row) on day 3. Magnification 10X. Scale bar = 100 µm. (c) Gene expression represented as fold change to Feeder Cells (FCs) of *CK-18* and *TGF-β* in hTCS (gray bars) and DhTCS (black bars) on day 15 (n = 2 replicates, n = 2 donors per condition) (Student’s t-test). (d) Representative images of DAPI (blue) nuclear stain and either CK-18 (red, left column) or TGF-β (red, right column) in hTCS (top row) and DhTCS (bottom row) on day 3. Magnification 10X. Scale bar = 100 µm. (e) Representative images of secondary only antibody for Alexa-Fluor 488 (green) and Alexa Fluor 555 (red) with Dapi stain (blue). Magnification 10X. Scale bar = 100 µm. **p* ≤ 0.05 to hTCS. Error bars represent standard deviation.


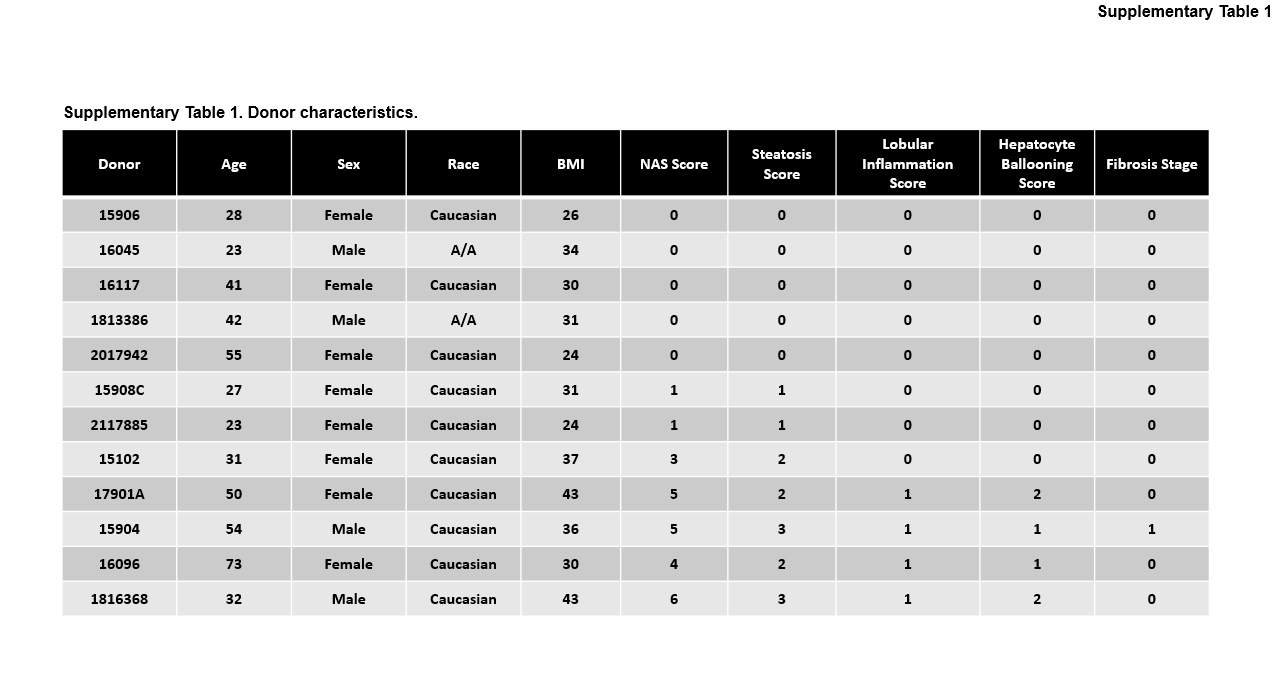


**Supplementary Table 1. Donor Characteristics.** Donor characteristics include age, sex, race, and BMI. Non-Alcoholic Fatty liver Disease (NAS) score, steatosis score, lobular inflammation score, hepatocyte ballooning score, and fibrosis stage are also listed.


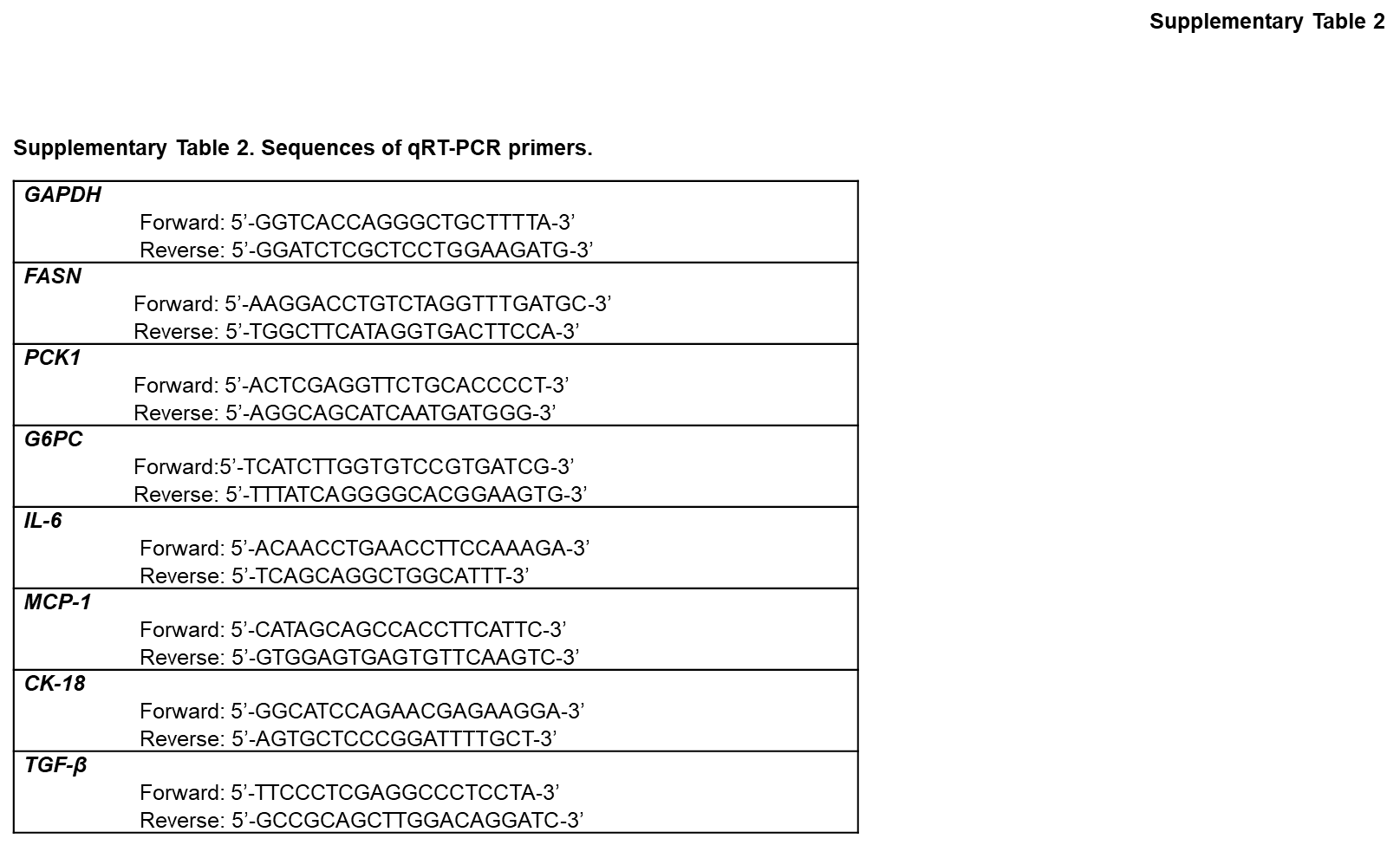


**Supplementary Table 2. Primer sequences for RT-PCR.** Primer sequences are listed for *Glyceraldehyde-3-phosphate dehydrogenase (GAPDH), Fatty Acid Synthase (FASN), Phosphoenolpyruvate Carboxykinase (PCK1), Glucose-6-Phosphate Catalytic subunit (G6PC), Interleukin-6 (IL-6), Monocyte Chemoattractant Protein-1 (MCP-1), Cytokeratin-18 (CK-18),* and *Transforming Growth Factor-β (TGF-β)* genes.
